# Increased P_I_O_2_ at Exhaustion in Hypoxia Enhances Muscle Activation and Swiftly Relieves Fatigue: A Placebo or a P_I_O_2_ Dependent Effect?

**DOI:** 10.3389/fphys.2016.00333

**Published:** 2016-08-17

**Authors:** Rafael Torres-Peralta, José Losa-Reyna, David Morales-Alamo, Miriam González-Izal, Ismael Pérez-Suárez, Jesús G. Ponce-González, Mikel Izquierdo, José A. L. Calbet

**Affiliations:** ^1^Department of Physical Education, University of Las Palmas de Gran CanariaLas Palmas, Spain; ^2^Research Institute of Biomedical and Health Sciences, Instituto Universitario de Investigaciones Biomédicas y SanitariasLas Palmas, Spain; ^3^Department of Health Sciences, Public University of NavarraTudela, Spain

**Keywords:** fatigue, performance, hypoxia, altitude, muscle activation, human experimentation, exercise, oxygenation

## Abstract

To determine the level of hypoxia from which muscle activation (MA) is reduced during incremental exercise to exhaustion (IE), and the role played by P_I_O_2_ in this process, ten volunteers (21 ± 2 years) performed four IE in severe acute hypoxia (SAH) (P_I_O_2_ = 73 mmHg). Upon exhaustion, subjects were asked to continue exercising while the breathing gas mixture was swiftly changed to a placebo (73 mmHg) or to a higher P_I_O_2_ (82, 92, 99, and 142 mmHg), and the IE continued until a new exhaustion. At the second exhaustion, the breathing gas was changed to room air (normoxia) and the IE continued until the final exhaustion. MA, as reflected by the *vastus medialis* (VM) and *lateralis* (VL) EMG raw and normalized root mean square (RMSraw, and RMSNz, respectively), normalized total activation index (TAINz), and burst duration were 8–20% lower at exhaustion in SAH than in normoxia (*P* < 0.05). The switch to a placebo or higher P_I_O_2_ allowed for the continuation of exercise in all instances. RMSraw, RMSNz, and TAINz were increased by 5–11% when the P_I_O_2_ was raised from 73 to 92, or 99 mmHg, and VL and VM averaged RMSraw by 7% when the P_I_O_2_ was elevated from 73 to 142 mmHg (*P* < 0.05). The increase of VM-VL average RMSraw was linearly related to the increase in P_I_O_2_, during the transition from SAH to higher P_I_O_2_ (*R*^2^ = 0.915, *P* < 0.05). In conclusion, increased P_I_O_2_ at exhaustion reduces fatigue and allows for the continuation of exercise in moderate and SAH, regardless of the effects of P_I_O_2_ on MA. At task failure, MA is increased during the first 10 s of increased P_I_O_2_ when the IE is performed at a P_I_O_2_ close to 73 mmHg and the P_I_O_2_ is increased to 92 mmHg or higher. Overall, these findings indicate that one of the central mechanisms by which severe hypoxia may cause central fatigue and task failure is by reducing the capacity for reaching the appropriate level of MA to sustain the task. The fact that at exhaustion in severe hypoxia the exercise was continued with the placebo-gas mixture demonstrates that this central mechanism has a cognitive component.

## Introduction

Muscle activation, as reflected by the root mean square of the electromyographic signal (EMG_*RMS*_), is higher in severe acute hypoxia (SAH) than normoxia at the same absolute intensity, but lower in hypoxia than in normoxia at the same relative intensity (Torres-Peralta et al., 2014). Close to exhaustion, the surface integrated electromyographic (iEMG) activity is higher during constant-intensity exercise in hyperoxia (F_I_O_2_ = 0.30) than in SAH (F_I_O_2_ = 0.10) (Amann et al., 2007). This could mean that hypoxia limits the motor drive output from the central nervous system (CNS) leading to reduced muscle activation (MA) and task failure. In agreement with this idea, during exercise in severe hypoxia, fatigue is rapidly relieved by oxygenation with normoxic (Calbet et al., 2003a) or hyperoxic gas (Amann et al., 2007). If hypoxia depresses muscle activation, oxygenation should be accompanied by an immediate increase in MA while the intensity of exercise remains at the same absolute level. However, it remains unknown whether the ergogenic effect of an increase in oxygenation requires a concomitant elevation of muscle activation.

During exercise in severe acute (Calbet et al., 2003a, 2015a; Amann et al., 2007; Morales-Alamo et al., 2015) and chronic hypoxia (Kayser et al., 1994; Calbet et al., 2003b) task failure is thought to be predominantly caused by central mechanisms sensitive to reduced O_2_ delivery to the brain (Goodall et al., 2012, 2014) and to reduced interstitial brain PO_2_ (Amann and Calbet, 2008). A fundamental difference between exercise in severe and moderate hypoxia is the region of the hemoglobin oxygen dissociation curve (ODC) at which the gas exchange occurs in the lungs. In severe hypoxia, pulmonary gas exchange occurs in the straight region of the ODC, implying that a small increase in arterial oxygen pressure (PaO_2_) would result in a greater elevation of arterial hemoglobin saturation (SaO_2_) (Calbet et al., 2003a; Calbet and Lundby, 2009). In moderate hypoxia, pulmonary gas exchange occurs at the upper and flatter region of the ODC, where an improvement in PaO_2_ translates into a smaller elevation of SaO_2_ (Amann et al., 2007). The fact that increasing inspiratory oxygen pressure (P_I_O_2_) to hyperoxic levels only relieved fatigue when applied at exhaustion in severe hypoxia could indicate that a substantial elevation of arterial oxygen content (CaO_2_) is required (Amann et al., 2007). However, the observation by Amann et al. (2007) that increased P_I_O_2_ does not relieve fatigue during moderate hypoxia could indicate that the increase in SaO_2_ is even more critical than the elevation of PaO_2_, since in the flatter region of the ODC the improvement of SaO_2_ for a given increase of PaO_2_ is smaller. It remains unknown what levels of improvement in PaO_2_ and CaO_2_ are required to relieve fatigue and enhance the neural activation of muscles upon exhaustion in hypoxia.

Therefore, the aims of this study were to (a) determine the influence of the level of hypoxia on a potential reduction of MA at exhaustion; (b) determine the minimum increase in P_I_O_2_ needed to enhance muscle activation at exhaustion in hypoxia; and (c) find out if the ergogenic effect of increasing P_I_O_2_ is always accompanied by enhanced muscle activation, which would be an indication of a predominantly central mechanism.

We hypothesized that an increase of P_I_O_2_ upon exhaustion would rapidly increase MA depending on the level of hypoxia at exhaustion and the inspiratory O_2_ pressure of the breathing gas.

## Materials and methods

### Subjects

Ten healthy men (age: 21.1 ± 2.1 years, height: 173 ± 8 cm, body mass: 71 ± 9 kg, body fat: 16.6 ± 4.5%, VO_2_max: 50.4 ± 4.7 mL.kg^−1^.min^−1^) agreed to participate in this investigation. After being informed about the experiments and the possible risks associated with participation they provided written consent. The study was performed by the Helsinki Declaration and was approved by the Ethical Committee of the University of Las Palmas de Gran Canaria (CEIH-2010-01 and CEIH-2009-01).

### General overview

This study was a part of a larger project that included several experiments designed to address the mechanisms limiting whole body exercise performance in humans. The results focusing on muscle metabolism and O_2_ transport have been published (Calbet et al., 2015a; Morales-Alamo et al., 2015). Body composition was determined by dual-energy x-ray absorptiometry (DEXA) (Hologic QDR-1500, Hologic Corp., software version 7.10, Waltham, MA), during the familiarization sessions. The leg muscle mass was calculated from the DEXA scans using the model of Wang et al. (1999). Subjects reported to the laboratory to familiarize with maximal exercise tests in normoxia and normobaric hypoxia (Altitrainer_200_, SMTEC, Switzerland) on separate days. For experimental purposes, subjects performed two sets of IE tests, here called *invasive* and *deception* test. On the first experimental day, all subjects performed the invasive tests as previously described (Calbet et al., 2015a) and on the second and third day, they completed the deception protocol. The exercise tests were carried out on a cycle ergometer (Lode Excalibur Sport 925900, Groningen, The Netherlands) and subjects were instructed to pedal at 80 revolutions per minute (rpm). To facilitate the maintenance of the targeted pedaling cadence, subjects received visual feedback, and verbal instructions when deviations of 5 or more rpm occurred.

### Exercise protocol

#### Invasive experiments

Subjects reported to the laboratory at 07.00 after an overnight fast from 22.00 h. After catheterization (see below), subjects were assigned to either an IE test to exhaustion in normoxia (30 W/2 min) or hypoxia (P_I_O_2_ = 73 mmHg; 20 W/2 min; Altitrainer200, SMTEC, Switzerland), in random order and separated by 90 min rest. Before the start of the IE in hypoxia, subjects were breathing the hypoxic gas for 3 min while they were pedaling 20–40 rpm with the ergometer unloaded. At exhaustion (Exh1), the subjects were rapidly switched to breath room air (normoxia) and requested to continue the exercise at the same load for 2 min, then the load was increased by 20 W every 2 min until exhaustion (Exh2). This was followed by a lunch break (a sandwich and 200 mL of apple or pineapple juice) and a 120 min resting period. Thereafter, the IE in hypoxia was repeated. At Exh1 the subjects were requested to keep pedaling while a valve deviated the inspired flow to a 30 L anesthesia bag pre-filled with hypoxic gas (F_I_O_2_ = ~13.3, P_*I*_O_2_ = ~91 mmHg) and a small amount of CO (7 mL·kg^−1^ body mass). The gas was breathed in an open circuit system in a well-ventilated room until the bag was almost emptied. The valve was then returned to the previous position such that the subjects continued the incremental test at this level of hypoxia (F_I_O_2_: ~13.3, P_I_O_2_: ~91 mmHg). After 2 min at the load eliciting exhaustion, the intensity was increased by 20 W/2 min until a new exhaustion (Exh2). Again, subjects were requested to keep pedaling while they were switched to breath room air (normoxia). After 2 min, the load was increased by 20 W/2 min until exhaustion (Exh3). The invasive experiments were used to study the influence of different levels of oxygenation on the hemodynamic responses and fatigue mechanisms in hypoxia, as reported previously (Calbet et al., 2015a).

#### Deception protocol (noninvasive)

**Figure 1:**
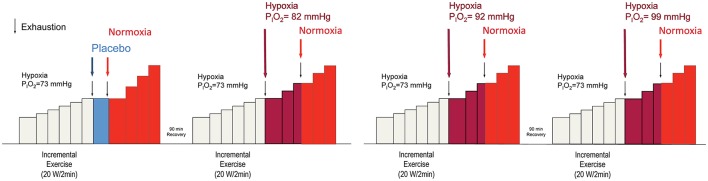
**Experimental protocol**. Each experimental day the subjects performed two incremental exercise tests in random order. The incremental exercise test always began in severe hypoxia (P_I_O_2_ = 73 mmHg). At exhaustion (Exh1) the breathing gas mixture was swiftly changed to another one with a greater oxygen PO_2_, except in one instance that the gas administered was the same one the subjects were breathing in severe acute hypoxia, i.e., P_I_O_2_ = 73 mmHg, to create a placebo condition. Subjects were asked to continue the exercise and after 2 min at the load eliciting Exh1, the load was increased by 20 or 30 W/2 min until exhaustion (Exh2). Once again, subjects were asked to keep pedaling while the gas mixture was swiftly changed to normoxia. After 2 min at the load eliciting Exh2, the intensity was increased by 20 or 30 W/2 min until the final exhaustion (Exh 3). Between Exh1 and Exh2, the breathing gas mixtures used corresponded to P_I_O_2_ of 73, 82, 92, 99, and 142 mmHg (Normoxia) and were administered following a double-blind design. Subjects were asked to pedal close to 80 rpm. However, when approaching exhaustion, the pedaling rate was always reduced.

Subjects performed four IE tests on 2 different days, separated by at least 1 week. A 90 min recovery period was established between the two tests carried out on the same day (Figure 1), as previously done (Calbet et al., 2003a). This resting period is sufficient to allow for a full recovery of peak power output and VO_2_max, as previously reported (Scharhag-Rosenberger et al., 2014; Calbet et al., 2015a). Each deception test was composed of an initial phase in severe hypoxia (P_*I*_O_2_ = 73 mmHg) (HYP1), followed by a second phase with a similar or a less severe level of hypoxia (HYP2), which continued with a final phase in normoxia (NX3). HYP1 started with an intensity of 60 or 70 W which, after 2 min was increased by 20 or 30 W every 2 min until exhaustion (Exh1). The 70 W starting load and the steps of 30 W were used in one of the subjects who was a well-trained triathlete, so the duration of his test was similar to the duration of the tests performed by the other subjects. Like during the invasive experiments, before the start of the IE in hypoxia, subjects were breathing the hypoxic gas for 3 min while they were pedaling at 20–40 rpm with the ergometer unloaded. At Exh1, the inspired gas mixture was rapidly changed to one of four different gas mixtures [P_*I*_O_2_ = 73 (placebo), 82, 92, and 99 mmHg, equivalent to 5200, 4400, 3600, and 3100 m above sea level, respectively]. Subjects were told and believed that they were getting normoxic gas at exhaustion. These gas mixtures were administered in random order and with a double-blind design. After 2 min at the load eliciting Exh1, the load was increased by 20 or 30 W every 2 min until exhaustion (Exh2). At Exh2, the gas mixture was rapidly changed to room air (P_*I*_O_2_ = 142 mmHg) while the subjects were strongly encouraged to continue pedaling. After 2 min at the load eliciting Exh2, the load was increased by 20 or 30 W every 2 min until exhaustion (Exh3). Although the change of P_I_O_2_ upon exhaustion was intended to be maintained for 2 min before increasing the load, in some instances, for example during the placebo experiments, subjects fatigued before reaching 2 min in the new oxygenation condition. In these cases, the breathing gas mixture was rapidly changed to normoxia, maintained for 2 min in normoxia, and then increased by 20 or 30 W every 2 min until exhaustion. Exhaustion during the IE tests was defined by either the subject stopping pedaling or dropping pedaling rate below 60 rpm during 5 s (or earlier if the cadence was dropping very fast), despite strong verbal encouragement. A 30 L anesthesia bag was prefilled with the target F_I_O_2_ and used as a buffer in the transition to HYP2, to gain few seconds to adjust the Altitrainer in such a way that the target F_I_O_2_ was instantaneously administered at the start of the transition. During the first 10–12 s of the transitions the subjects breathed from the anesthesia bag, then a four-way valve was used to direct the inspiratory port to either the Altitrainer or room air. These 10 s (bag breathing) were used to stabilize the Altitrainer at the target F_I_O_2_ corresponding to each HYP2 phase.

### Oxygen uptake and hemoglobin oxygen saturation

Oxygen uptake was measured with a metabolic cart (Vmax N29; Sensormedics, California, USA), calibrated before each test according to the manufacturer instructions. Respiratory variables were analyzed breath-by-breath and averaged every 10 s for the analysis of transitions at exhaustion. Hemoglobin oxygen saturation was estimated with a finger pulse oximeter (SpO_2_) (OEM III module, 4549-000, Plymouth, MN).

### Electromyography

**Figure 2:**
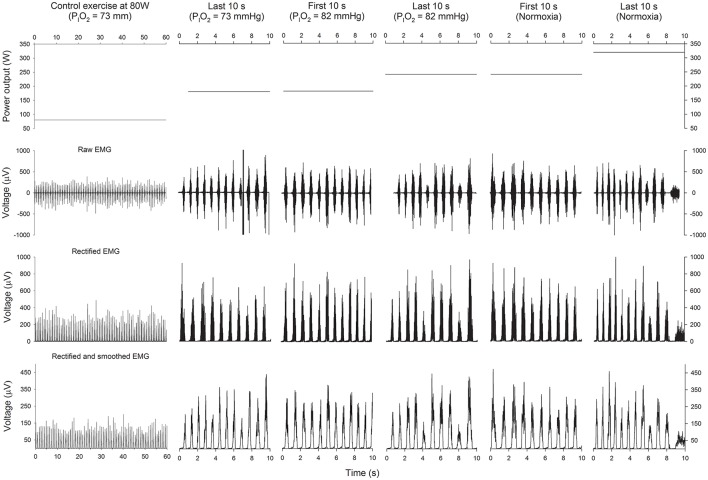
**Power output and EMG**. Schematic representation of the power output (upper panels), raw EMG (2nd row), rectified EMG (3th row), and rectified and smoothed EMG (lower panels), during the last 60 s of the control submaximal exercise at 80 W in hypoxia (P_I_O_2_ = 73 mmHg), the last 10 s of the incremental exercise (IE) in severe hypoxia (P_I_O_2_ = 73 mmHg), the first 10 s of the transition from a P_I_O_2_ of 73 to 82 mmHg, the last 10 s of the IE at a P_I_O_2_ of 82 mmHg, the first 10 s in normoxia and the last 10 s before task failure in normoxia.

Electrical MA was monitored using surface electromyography (EMG) (Figure 2). EMG signals were continuously recorded from the *vastus medialis* and *vastus lateralis*, as previously reported (Torres-Peralta et al., 2016). Before the application of the EMG electrodes, the skin surface was carefully shaved, and wiped with ethanol to reduce skin impedance. Bipolar single differential electrodes were placed longitudinally on the muscles following the SENIAM recommendations (Merletti and Hermens, 2000) and taped on the skin to minimize movement artifacts. The reference electrode was placed on the skin over the acromion. The position of the electrodes was marked on the skin with indelible ink, and these references were used for precise electrode placement in repeated experiments.

The EMG signals were acquired using a 16-channel recording system (Myomonitor IV, Delsys Inc., Boston, MA) at a sampling rate of 1000 Hz using rectangular shaped (19.8 mm wide and 35 mm long) bipolar surface electrodes with 1 × 10 mm 99.9% Ag conductors, and with an inter-conductor distance of 10 mm (DE-2.3 Delsys Inc.). The EMG data were filtered with a high-pass filter of 20 Hz and a low-pass filter of 450 Hz using a fifth-order Butterworth filter. The system has an input impedance of >10^15^Ω per 0.2pF of input capacitance, a common mode rejection ratio of >80 dB, signal-to-noise ratio < 1.2 μV, and a pre-amplifier gain 1000 V/V ±1 %. Each pedal revolution was detected using an electrogoniometer (Goniometer Biosignal Sensor S700 Joint Angle Shape Sensor; Delsys Inc. Boston) fixed on the left knee and sampled at 500 Hz. The electrogoniometer was individually calibrated taking as references the knee angles in fully extended and flexed positions. EMG and joint movement were simultaneously recorded by a portable device (Myomonitor IV, Delsys Inc. Boston) and wirelessly transmitted to a computer (EMGWorks Wireless application and EMGWorks Acquisition 3.7.1.3; Delsys, Inc. Boston).

The EMG signal corresponding to each muscle contraction was analyzed using code developed “in house” (Matlab R2012b, MathWorks, Natick, MA, USA). The EMG recordings were full-wave rectified and smoothed to provide an index of muscle activation; the amplitude characteristics were analyzed via average RMS of a 25-ms moving window for the duration of the contraction burst. Contraction burst onset and offset detection were determined using 20% of the maximal EMG_RMS_ activity of each contraction burst as a reference (Baum and Li, 2003; Hug and Dorel, 2009; Torres-Peralta et al., 2014), rather than a mean threshold value from 15 consecutive contraction bursts (Ozgunen et al., 2010). This approach yielded the same result as direct, simple visual discrimination, with 100% detection of all contraction bursts. Contraction timing was defined as the time elapsed from the knee at is greatest extension to the start of the contraction burst, expressed as a percentage of the full duration of each revolution. The EMG_RMS_ recorded during the last minute of a 2 min 80 W load (in hypoxia, P_I_O_2_ = 73 mmHg) was used to normalize the remaining EMG_RMS_ data. Besides, we defined a total activity index (TAI) as TAI = EMG_RMS_ × burst duration (ms) × number of pedal strokes during the period of time analyzed. The total activity index is similar to the integrated EMG signal, but was computed separately for each contraction burst and excluded the baseline EMG between contraction bursts (Torres-Peralta et al., 2014). The TAI recorded during the last minute of a 2 min 80 W load (in hypoxia) was used to normalize the rest of the TAI values.

The mean (MPF) and median (MdPF) power spectrum frequencies were calculated using Fast Fourier Transform (Solomonow et al., 1990). All variables were reported as the mean values of the pedal strokes recorded during the last 10 and 30 s of the incremental exercise. EMG data are reported separately for *vastus medialis* (VM) and *lateralis* (VL), and also as the average of the two muscles.

### Calculation of the improvement in SaO_2_ during the first 10 s of the transitions

The mean change in SaO_2_ needed to explain the mean improvement in VO_2_ observed during the first 10 s of the transition from hypoxia to higher a P_I_O_2_ was calculated by solving the Fick equation, using arterial blood gasses and thermodilution cardiac output data obtained in normoxia and hypoxia (P_*I*_O_2_ = 73 mmHg) in parallel invasive experiments performed by the same subjects (Calbet et al., 2015a). Since similar levels of peak cardiac output were reached in severe hypoxia and normoxia, it was assumed that the level of cardiac output reached at exhaustion at intermediate P_I_O_2_ levels (i.e., 82, 92, and 99 mmHg) must have been similar to that measured in normoxia. It was also assumed that cardiac output remained unchanged during the first 10 s of the transition, given the stability of heart rate during the transitions and the high dependency of cardiac output on the absolute exercise intensity (Calbet and Lundby, 2009; Calbet et al., 2009a, 2015b), which remained unchanged during the first 10 s of the transition.

### Statistics

Normal distribution of variables was checked using the Shapiro-Wilks test. Since variables were normally distributed, differences between tests at Exh1 were determined using one-way repeated measures analysis of variance (ANOVA). The Mauchly's test of sphericity was run before the ANOVA and in the case of violation of the sphericity assumption the degrees of freedom were adjusted according to the Huynh and Feldt test. Pairwise comparisons at specific time points were performed with Student's paired *t*-tests and adjusted for multiple comparisons with the Holm–Bonferroni method. Since no significant differences were observed at exhaustion between the four tests in severe hypoxia (P_I_O_2_ = 73 mmHg), these four tests were averaged to obtain a representative value for exhaustion at a P_I_O_2_ of 73 mmHg. The same procedure was used to test for differences between the four IE tests ending in normoxia (Exh3). Similar results were obtained in the four tests at exhaustion in normoxia (Exh3) and hence, the values obtained in these four tests were also averaged to generate a single value representing normoxia. These two averages were compared with Student's paired *t*-tests. The effect of increasing P_I_O_2_ at exhaustion on all dependent variables was assessed using a two-way ANOVA for repeated measures with two factors: breathing gas (two levels: pre- vs. post-switch to the new breathing gas) and P_I_O_2_ (four levels), followed by pairwise comparisons using Student's paired *t*-tests adjusted for multiple comparisons with the Holm–Bonferroni method. The relationships between changes in P_I_O_2_ and the changes in the dependent variables were tested using linear regression analysis. To compare the first 10 s of the transition between the first and the second transition, an average value for the four conditions of each transition was calculated. This generated a single value per subject for the first and another unique value per subject for the second transition. The two transitions were compared with a paired Student's *t*-test. Values are reported as the mean ± standard deviation (unless otherwise stated). *P* ≤ 0.05 was considered statistically significant. All statistical analyses were performed using SPSS v.15.0 for Windows (SPSS Inc., Chicago, IL) and Excel 2011 (Microsoft, Redmond, WA, USA).

## Results

### Maximal exercise in severe acute hypoxia (P_I_O_2_ = 73 mmHg) and normoxia (P_I_O_2_ = 142 mmHg)

As shown in Table 1, SpO_2_, power output at exhaustion (Wmax), VO_2_peak, pulmonary ventilation at exhaustion (V_*E*_), respiratory rate (RR), heart rate at exhaustion (HR), end-tidal O_2_ pressure (P_*ET*_O_2_), end-tidal CO_2_ pressure (P_*ET*_CO_2_), and carbon dioxide production (VCO_2_) were lower during the last 30 s of exercise in severe hypoxia than in normoxia, while the respiratory exchange ratio (RER) was higher in hypoxia than in normoxia (all *P* ≤ 0.05).

**Table 1:** **Ergospirometric and electromyographic responses during the last 30 s of the incremental exercise to exhaustion in normoxia (P_I_O_2_ ≈ 142 mmHg) and severe hypoxia (P_I_O_2_ ≈ 73 mmHg)**.

	**Hypoxia (P_I_O_2_ = 73 mmHg)**	**Normoxia**	***P***
F_I_O_2_ (%)	10.8 ± 0.07	20.8 ± 0.04	< 0.001
SpO_2_ (%)	63.8 ± 5.7	92.8 ± 3.1	< 0.001
Wmax (W)	170.5 ± 17.9	213 ± 19.7	< 0.001
VO_2_peak (L.min^−1^)	2.28 ± 0.19	3.44 ± 0.43	< 0.001
V_E_ (L.min^−1^)	115.2 ± 18.6	124.8 ± 15.6	< 0.001
RR (breaths.min^−1^)	50.6 ± 7.0	55.9 ± 7.1	< 0.001
HR (beats.min^−1^)	179.0 ± 8.4	184.8 ± 5.2	< 0.001
P_*ET*_O_2_ (mmHg)	51.3 ± 2.3	108.2 ± 7.4	< 0.001
P_*ET*_CO_2_ (mmHg)	28.1 ± 2.5	30.8 ± 3.1	< 0.001
RER	1.34 ± 0.13	1.05 ± 0.06	< 0.001
VCO_2_ (L.min^−1^)	3.06 ± 0.36	3.55 ± 0.41	< 0.001
RPM	71.9 ± 4.1	68.4 ± 4.2	0.08
VM RMSraw (μV)	111.2 ± 38.6	128.3 ± 42.4	< 0.01
VL RMSraw (μV)	97.5 ± 30.8	110.4 ± 28.0	< 0.01
Average RMSraw (μV)	104.4 ± 29.0	119.4 ± 28.8	< 0.005
VM RMSNz (A.U.)	178.1 ± 35.2	209.2 ± 58.4	< 0.05
VL RMSNz (A.U.)	173.3 ± 35.4	200.0 ± 49.1	< 0.005
Average RMSNz (A.U.)	175.4 ± 31.2	204.6 ± 50.3	< 0.01
VM TAINz (A.U.)	111.5 ± 33.5	138.7 ± 47.8	< 0.005
VL TAINz (A.U.)	97.3 ± 21.0	117.4 ± 22.7	< 0.001
Average TAINz (A.U.)	102.9 ± 25.5	126.7 ± 30.9	< 0.001
VM MPF (Hz)	89.8 ± 16.9	85.2 ± 16.6	< 0.001
VL MPF (Hz)	89.6 ± 16.5	85.5 ± 17.1	< 0.001
Average MPF (Hz)	89.7 ± 16.7	85.4 ± 16.9	< 0.001
VM MdPF (Hz)	71.3 ± 12.1	69.2 ± 11.8	0.06
VL MdPF (Hz)	70.6 ± 12.1	68.9 ± 12.2	0.06
Average MdPF (Hz)	70.9 ± 12.1	69.0 ± 12.0	0.06
VM Burst (ms)	305.4 ± 51.5	334.3 ± 34.8	< 0.05
VL Burst (ms)	283.0 ± 34.5	306.2 ± 26.7	< 0.05
Average Burst (ms)	294.2 ± 42.1	320.2 ± 29.6	< 0.05

*F_I_O_2_, inspiratory oxygen fraction; SpO_2_, hemoglobin saturation in capillary blood measured by pulse oximetry; Wmax, power output at exhaustion; VO_2_, oxygen consumption; V_E_, pulmonary ventilation; RR, respiratory rate; HR, heart rate; P_ET_O_2_, end-tidal O_2_ pressure; P_ET_CO_2_, end-tidal CO_2_ pressure; RER, respiratory exchange ratio; VCO_2_, CO_2_ production; RPM, revolutions per minute; VL, vastus lateralis; VM, vastus medialis; RMSraw, raw root mean square; RMSNz, normalized root mean square; TAINz, normalized total activation index (arbitrary units, A.U.); MPF, mean power frequency; MdPF, median power frequency; Burst, contraction burst duration. n = 10*.

Muscle activation, as reflected by VM and VL raw and normalized RMS, total activation index and contraction burst duration was 8–20% lower in hypoxia than normoxia (*P* < 0.05) (Table 1). In contrast, MPF was 5% lower in normoxia than hypoxia (*P* < 0.001) and a similar trend was observed for MdPF (Table 1).

### Effect of increased P_I_O_2_ on cardiorespiratory and EMG variables

**Figure 3:**
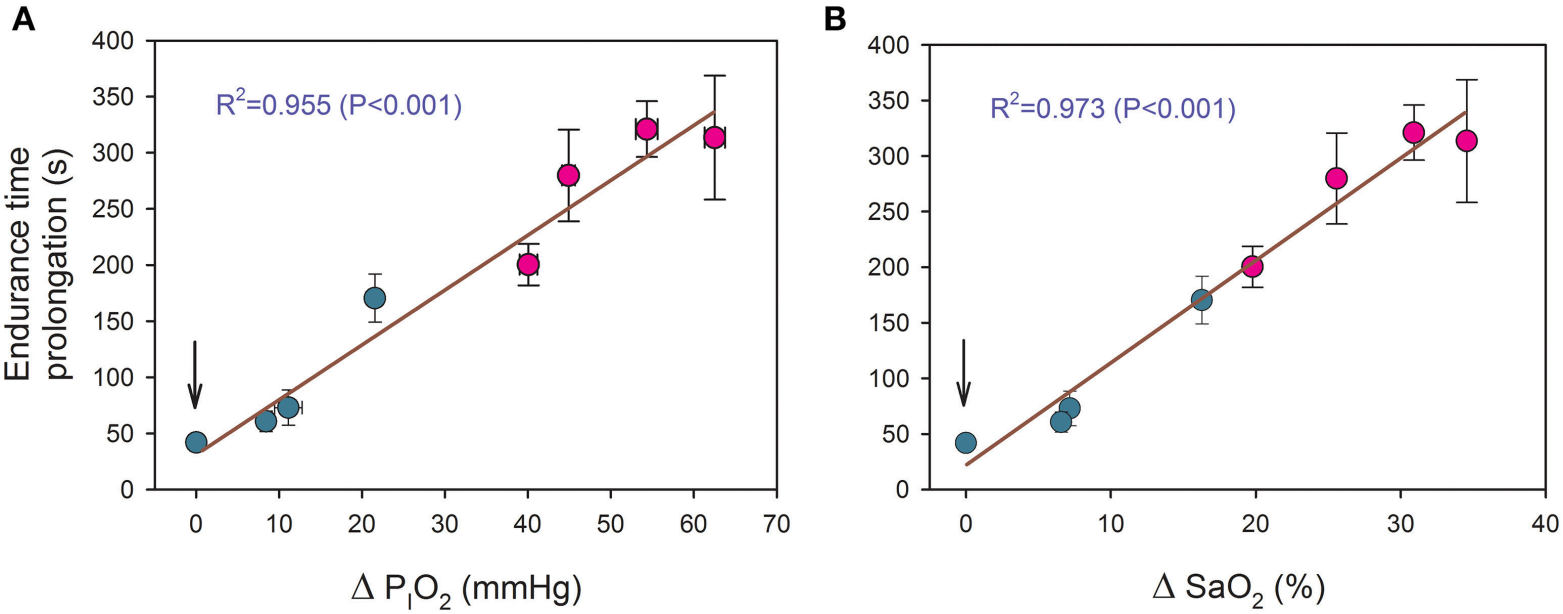
**Relationship between the duration of new oxygenation phases with: (A) the increase of P_**I**_O_**2**_ (ΔP_**I**_O_**2**_) and (B) the estimated improvement in arterial saturation (ΔSaO_**2**_)**. Green circles: transitions from severe hypoxia (P_I_O_2_ = 73 mmHg) to placebo (vertical arrow) and moderate hypoxia; red circles: transitions from different levels of hypoxia (P_I_O_2_ of 73, 82, 92, and 99 mmHg) to normoxia. Each point corresponds to the mean of 10 subjects.

Increased P_I_O_2_ allowed for the continuation of exercise during 41.9 ± 19.8, 60.7 ± 30.2, 72.9 ± 52.0, and 170.5 ± 70.8 s for the transition from a P_I_O_2_ of 73 mmHg to placebo, 82, 92, and 99 mmHg, respectively, (all *P* < 0.05, compared to the end exercise in severe acute hypoxia). There was a linear relationship between the duration of the new oxygenation phases and the increase of P_I_O_2_ (time (s) = 35.1+ 4.86· ΔP_I_O_2_; *R*^2^ = 0.955, *P* < 0.001, *n* = 8), where ΔP_I_O_2_ represents the increase in P_I_O_2_ in mmHg (Figure 3A). A similar relationship was obtained between endurance time and the estimated improvement in SaO_2_ (time (s) = 21.1+ 9.17· ΔS_a_O_2_; *R*^2^ = 0.973, *P* < 0.001, *n* = 8) (Figure 3B).

Compared to the mean values observed during the last 10 s of exercise in severe hypoxia (P_I_O_2_ = 73 mmHg), P_ET_O_2_, and VO_2_ were increased, and RER reduced during the first 10 s following the increase in oxygenation (Tables 2 and 3). SpO_2_ was only significantly increased in transitions to normoxia (Table 3), in part due to the slow response time of the pulse oximeter. These effects were more accentuated the greater the difference in P_I_O_2_ between the hypoxic and the increased P_I_O_2_ condition.

**Table 2:** **Cardiorespiratory responses during the last 10 s of an incremental exercise to exhaustion in severe hypoxia (P_**I**_O_**2**_ = 73 mmHg) and during the first 10 s of oxygenation with different gas mixtures**.

	**Exhaustion P_I_O_2_ = 73 mmHg**	**Start of P_I_O_2_ = 99 mmHg**	**Exhaustion P_I_O_2_ = 73 mmHg**	**Start of P_I_O_2_ = 92 mmHg**	**Exhaustion P_I_O_2_ = 73 mmHg**	**Start of P_I_O_2_ = 82 mmHg**	**Exhaustion P_I_O_2_ = 73 mmHg**	**Start of P_I_O_2_ = 73 mmHg**
F_I_O_2_ (%)	10.78 ± 0.10	13.92 ± 0.23^c^	10.82 ± 0.12	12.45 ± 0.75^c^	10.78 ± 0.06	12.01 ± 0.25^c^	10.79 ± 0.08	10.79 ± 0.20^¶^^§^^‡^
SpO_2_ (%)	62.4 ± 5.2	63.1 ± 5.5	64.3 ± 5.5	65.1 ± 5.7	63.5 ± 5.8	63.6 ± 5.9	64.7 ± 4.9	64.4 ± 5.7^‡^
Wmax (W)	172.0 ± 23.5	172.0 ± 23.5	170.0 ± 21.6	170.0 ± 21.6	168.0 ± 16.9	168.0 ± 16.9	172.0 ± 21.5	172.0 ± 21.5
VO_2_peak (L.min^−1^)	2.32 ± 0.17	2.81 ± 0.46^b^	2.23 ± 0.25	2.53 ± 0.34^a^	2.33 ± 0.15	2.45 ± 0.27^T^	2.27 ± 0.29	2.29 ± 0.32^¶^^§^^‡^
V_*E*_ (L.min^−1^)	118.2 ± 23.7	111.8 ± 20.8	114.8 ± 26.3	116.8 ± 22.6	117.5 ± 16.4	116.4 ± 15.1	114.8 ± 14.7	116.8 ± 15.6
RR (breaths.min^−1^)	51.7 ± 9.2	48.1 ± 7.2	51.3 ± 8.5	50.7 ± 8.2	51.5 ± 8.4	51.1 ± 7.6	51.3 ± 7.0	52.3 ± 7.7
HR (beats.min^−1^)	179.0 ± 10.2	179.3 ± 10.1	180.5 ± 8.2	181.0 ± 7.4	177.3 ± 7.4	177.5 ± 7.2	180.6 ± 7.9	180.8 ± 8.3^¶^
P_*ET*_O_2_ (mmHg)	51.4 ± 3.0	62.9 ± 5.1^c^	51.7 ± 3.1	58.1 ± 6.3^c^	51.4 ± 2.9	56.1 ± 3.9^c^	51.6 ± 2.0	51.9 ± 2.0^¶^^§^^‡^
P_*ET*_CO_2_ (mmHg)	27.3 ± 2.9	27.3 ± 4.3	28.3 ± 2.9	28.4 ± 2.7	27.4 ± 3.7	27.6 ± 3.0	28.5 ± 2.3	28.2 ± 2.4
RER	1.32 ± 0.17	1.16 ± 0.15^T^	1.36 ± 0.15	1.28 ± 0.13	1.32 ± 0.15	1.28 ± 0.15	1.36 ± 0.14	1.37 ± 0.17^§^
VCO_2_ (L.min^−1^)	3.06 ± 0.42	3.01 ± 0.44	3.03 ± 0.48	3.12 ± 0.41	3.07 ± 0.29	3.06 ± 0.28	3.08 ± 0.41	3.11 ± 0.39
RPM	63.6 ± 9.4	66.3 ± 10.3	68.5 ± 7.0	71.3 ± 11.4	67.1 ± 9.5	68.2 ± 12.0	71.2 ± 7.8	70.0 ± 9.3
VM RMSraw (μV)	105.9 ± 37.2	119.3 ± 43.5^c^	106.3 ± 42.6	112.2 ± 38.1^a^	113.5 ± 42.7	120.6 ± 50.2^T^	97.2 ± 43.0	99.7 ± 42.6^¶^^‡^
VL RMSraw (μV)	108.8 ± 38.4	119.1 ± 39.9^c^	97.3 ± 48.9	102.0 ± 49.2^a^	115.8 ± 49.9	114.1 ± 45.1	85.9 ± 35.5	87.4 ± 40.6^¶^
Average RMSraw (μV)	107.4 ± 29.1	119.2 ± 33.4^c^	101.8 ± 41.5	107.1 ± 39.1^b^	114.7 ± 39.2	117.3 ± 40.6	91.5 ± 34.9	93.6 ± 38.0^¶^^‡^
VM RMSNz (A.U.)	176.2 ± 48.1	195.8 ± 56.9^c^	178.0 ± 58.4	191.2 ± 57.2^a^	180.9 ± 50.4	187.6 ± 41.7	164.8 ± 67.2	168.6 ± 64.2^¶^
VL RMSNz (A.U.)	182.5 ± 55.5	199.6 ± 52.2^c^	160.2 ± 38.8	171.1 ± 35.5^a^	189.6 ± 67.0	185.6 ± 45.4	151.7 ± 49.2	152.3 ± 51.7^¶^^‡^
Average RMSNz (A.U.)	179.4 ± 49.8	197.7 ± 52.5^c^	169.1 ± 47.0	181.2 ± 45.0^a^	185.2 ± 56.4	186.6 ± 40.2	158.2 ± 56.8	160.5 ± 56.9^¶^^‡^
VM TAINz (A.U.)	39.6 ± 17.6	44.5 ± 19.5^b^	35.6 ± 14.8	38.0 ± 12.5	37.1 ± 14.1	37.6 ± 11.1	33.5 ± 13.3	35.5 ± 14.1^¶^
VL TAINz (A.U.)	36.8 ± 13.5	41.2 ± 14.4^c^	30.2 ± 9.7	32.8 ± 7.1	37.1 ± 12.6	35.8 ± 8.7	29.3 ± 8.8	31.6 ± 14.0^¶^
Average TAINz (A.U.)	38.2 ± 15.4	42.8 ± 16.9^c^	32.9 ± 11.8	35.4 ± 9.5	37.1 ± 13.2	36.7 ± 9.6	31.4 ± 10.6	33.6 ± 13.3^¶^
VM MPF (Hz)	96.0 ± 24.8	95.1 ± 24.2	89.2 ± 22.8	91.5 ± 23.9	91.2 ± 17.3	89.8 ± 15.0	84.9 ± 15.5	83.6 ± 13.8
VL MPF (Hz)	97.0 ± 27.0	96.0 ± 27.5	88.9 ± 22.6	90.2 ± 23.2	91.2 ± 17.3	89.5 ± 15.5	84.9 ± 15.9	83.5 ± 14.4
Average MPF (Hz)	96.5 ± 25.9	95.5 ± 25.7	89.1 ± 22.7	90.9 ± 23.5	91.2 ± 17.3	89.6 ± 15.3	84.9 ± 15.7	83.5 ± 14.1
VM MdPF (Hz)	76.3 ± 17.1	76.9 ± 17.1	70.3 ± 15.7	72.7 ± 14.7	70.8 ± 13.2	71.8 ± 11.6	66.9 ± 10.3	66.2 ± 10.5
VL MdPF (Hz)	77.8 ± 19.7	77.6 ± 20.0	69.8 ± 15.9	70.9 ± 15.0	70.7 ± 13.2	71.4 ± 12.0	66.4 ± 10.8	65.6 ± 10.2
Average MdPF (Hz)	77.0 ± 18.3	77.3 ± 18.4	70.0 ± 15.8	71.8 ± 14.7	70.8 ± 13.2	71.6 ± 11.7	66.7 ± 10.5	65.9 ± 10.3^§^
VM Burst (ms)	361.5 ± 110.1	349.9 ± 85.9	310.7 ± 88.3	305.1 ± 81.2	310.3 ± 62.5	304.9 ± 94.7	309.3 ± 80.7	318.2 ± 74.7
VL Burst (ms)	343.2 ± 91.7	324.8 ± 81.7	297.8 ± 55.6	300.1 ± 70.7	310.2 ± 59.7	303.9 ± 91.5	300.0 ± 63.7	316.5 ± 86.6
Average Burst (ms)	352.4 ± 95.3	337.3 ± 79.0	304.3 ± 71.3	302.6 ± 75.4	310.2 ± 60.8	304.4 ± 93.0	304.7 ± 71.0	317.4 ± 76.5
VM Timing (%)	48.8 ± 3.8	47.6 ± 4.2^a^	49.4 ± 2.5	49.1 ± 3.2	49.4 ± 2.3	49.3 ± 2.9	47.9 ± 3.9	48.3 ± 4.2^‡^
VL Timing (%)	50.7 ± 2.1	50.0 ± 2.3	50.2 ± 2.3	49.9 ± 2.5	50.3 ± 2.1	50.3 ± 2.4	49.5 ± 2.9	49.6 ± 3.0
Average Timing (%)	49.7 ± 2.6	48.8 ± 2.7	49.8 ± 2.3	49.5 ± 2.7	49.8 ± 2.2	49.8 ± 2.6	48.7 ± 3.4	49.0 ± 3.5

*F_I_O_2_, inspiratory oxygen fraction; SpO_2_, hemoglobin saturation in capillary blood measured by pulse oximetry; Wmax, power output at exhaustion; VO_2_, oxygen consumption; V_E_, pulmonary ventilation; RR, respiratory rate; HR, heart rate; P_ET_O_2_, end-tidal O_2_ pressure; P_ET_CO_2_, end-tidal CO_2_ pressure; RER, respiratory exchange ratio; VCO_2_, CO_2_ production; RPM, revolutions per minute; VL, vastus lateralis; VM, vastus medialis; RMSraw, raw root mean square; RMSNz, normalized root mean square; TAINz: normalized total activation index (arbitrary units, A.U.); MPF, mean power frequency; MdPF, median power frequency; Burst, contraction burst duration; Timing: start of activation expressed as percentage of total revolution duration*.

a *P < 0.05*;

b *P < 0.01*;

c P < 0.001; and

T *P < 0.1 (F_I_O_2_ = 73 mmHg vs. new gas mixture)*.

¶ *P < 0.05 ANOVA breathing gas switch main effect*;

§ *P < 0.05 ANOVA oxygenation level main effect*;

‡ *P < 0.05 ANOVA breathing gas switch x oxygenation level interaction; n = 10*.

**Table 3:** **Cardiorespiratory responses during the last 10 s of an incremental exercise to exhaustion in different levels of hypoxia (P_***I***_O_**2**_ = 73, 82, 92, and 99 mmHg) and during the first 10 s of oxygenation to normoxia (P_**I**_O_**2**_ = 142 mmHg)**.

	**Exhaustion P_I_O_2_ = 99 mmHg**	**Start of Normoxia**	**Exhaustion P_I_O_2_ = 92 mmHg**	**Start of Normoxia**	**Exhaustion P_I_O_2_ = 82 mmHg**	**Start of Normoxia**	**Exhaustion P_I_O_2_ = 73 mmHg**	**Start of Normoxia**
F_I_O_2_ (%)	14.42 ± 0.12	20.26 ± 0.46^c^	13.41 ± 0.39	19.99 ± 0.53^c^	11.98 ± 0.21	19.90 ± 0.62^c^	10.91 ± 0.44	20.02 ± 0.74^c^^¶^^§^^‡^
SpO_2_ (%)	78.2 ± 4.1	80.2 ± 5.3^a^	70.4 ± 6.7	73.4 ± 6.8^c^	67.6 ± 4.5	68.2 ± 4.2^b^	64.7 ± 4.8	66.3 ± 6.20^a^^¶^^§^^‡^
Wmax (W)	180.0 ± 21.1	180.0 ± 21.1	172.0 ± 19.3	172.0 ± 19.3	170.0 ± 17.0	170.0 ± 17.0	172.0 ± 21.5	172.0 ± 21.5
VO_2_peak (L.min^−1^)	2.93 ± 0.25	3.89 ± 0.68^b^	2.87 ± 0.28	4.15 ± 0.45^c^	2.48 ± 0.32	3.99 ± 0.52^c^	2.37 ± 0.30	4.05 ± 0.59^c^^¶^^§^^‡^
V_*E*_ (L.min^−1^)	119.9 ± 18.1	116.1 ± 25.6	118.6 ± 19.0	117.3 ± 16.7	110.8 ± 24.5	109.5 ± 17.8	117.6 ± 17.5	113.6 ± 21.2
RR (breaths.min^−1^)	53.5 ± 7.1	51.2 ± 5.7	53.2 ± 7.0	52.6 ± 6.0	49.6 ± 8.6	49.5 ± 5.6	52.7 ± 7.2	52.2 ± 7.7
HR (beats.min^−1^)	182.2 ± 7.9	182.1 ± 8.7	182.7 ± 6.2	182.9 ± 6.2	178.6 ± 7.0	178.5 ± 6.5	180.9 ± 7.9	180.5 ± 7.9
P_*ET*_O_2_ (mmHg)	71.3 ± 2.6	96.4 ± 9.9^c^	64.4 ± 3.1	90.6 ± 13.4^c^	56.7 ± 3.4	91.4 ± 12.2^c^	51.8 ± 2.1	92.3 ± 11.5^c^^¶^^§^^‡^
P_*ET*_CO_2_ (mmHg)	29.0 ± 2.9	30.5 ± 3.0^a^	29.0 ± 2.9	30.1 ± 2.5^a^	28.7 ± 3.6	29.5 ± 3.5^a^	28.3 ± 2.4	30.2 ± 2.7^a^
RER	1.13 ± 0.08	0.92 ± 0.10^c^	1.12 ± 0.08	0.87 ± 0.12^c^	1.21 ± 0.11	0.84 ± 0.10^c^	1.35 ± 0.14	0.90 ± 0.15^c^^¶^^§^^‡^
VCO_2_ (L.min^−1^)	3.28 ± 0.28	3.29 ± 0.51	3.21 ± 0.34	3.25 ± 0.29	3.00 ± 0.47	3.02 ± 0.37	3.13 ± 0.40	3.16 ± 0.41^§^
RPM	61.7 ± 9.0	67.0 ± 12.5	63.8 ± 11.4	69.5 ± 10.2^b^	61.9 ± 11.3	68.1 ± 9.4^T^	58.3 ± 12.6	65.6 ± 13.9^T^^¶^
VM RMSraw (μV)	116.9 ± 45.3	122.7 ± 46.6	120.2 ± 42.5	119.8 ± 39.2	120.1 ± 49.4	125.3 ± 47.6	102.6 ± 47.0	108.4 ± 44.3
VL RMSraw (μV)	116.5 ± 38.1	123.6 ± 43.3	107.3 ± 52.5	110.8 ± 52.6	118.4 ± 50.2	122.7 ± 52.9	87.2 ± 36.1	94.5 ± 37.0^b^^¶^
Average RMSraw (μV)	116.7 ± 32.5	123.2 ± 35.9	113.8 ± 42.6	115.3 ± 40.3	119.3 ± 41.6	124.0 ± 43.1	94.9 ± 37.5	101.5 ± 36.3^a^^¶^
VM RMSNz (A.U.)	182.1 ± 53.9	191.7 ± 57.8	204.3 ± 70.8	205.8 ± 68.7	189.5 ± 57.3	197.9 ± 51.7	172.9 ± 70.7	184.7 ± 77.1^¶^
VL RMSNz (A.U.)	195.4 ± 52.6	206.8 ± 66.6	177.8 ± 41.3	183.4 ± 43.3	194.5 ± 70.0	199.4 ± 57.5	155.9 ± 55.7	167.3 ± 53.4^a^^¶^
Average RMSNz (A.U.)	188.7 ± 43.9	199.2 ± 52.7	191.1 ± 53.8	194.6 ± 50.8	192.0 ± 61.4	198.6 ± 51.6	164.4 ± 62.0	176.0 ± 63.9^¶^
VM TAINz (A.U.)	39.9 ± 15.7	43.6 ± 16.2^T^	42.8 ± 19.2	40.6 ± 11.2	42.1 ± 17.0	42.6 ± 14.5	36.1 ± 14.4	38.0 ± 16.4^¶^
VL TAINz (A.U.)	43.2 ± 19.3	42.4 ± 14.4	34.1 ± 9.0	35.3 ± 7.2	40.5 ± 15.2	40.9 ± 12.8	32.5 ± 13.8	34.1 ± 15.6
Average TAINz (A.U.)	41.5 ± 17.1	43.0 ± 15.1	38.4 ± 13.4	37.9 ± 8.4	41.3 ± 15.9	41.7 ± 13.5	34.3 ± 13.5	36.0 ± 15.5^¶^
VM MPF (Hz)	87.4 ± 20.1	84.9 ± 20.6	84.7 ± 20.2	86.0 ± 21.0	88.5 ± 14.6	87.0 ± 16.6	81.8 ± 15.2	81.7 ± 16.4
VL MPF (Hz)	71.4 ± 15.8	85.3 ± 21.9^c^	85.7 ± 19.4	85.6 ± 21.8	88.1 ± 15.0	86.3 ± 16.9	81.9 ± 15.5	82.1 ± 16.1^¶^^‡^
Average MPF (Hz)	79.4 ± 17.8	85.1 ± 21.2^b^	85.2 ± 19.8	85.8 ± 21.4	88.3 ± 14.8	86.6 ± 16.7	81.8 ± 15.3	81.9 ± 16.2^‡^
VM MdPF (Hz)	70.9 ± 16.2	69.9 ± 16.2	67.5 ± 13.6	68.7 ± 15.2	72.1 ± 11.8	70.3 ± 12.2	65.0 ± 10.6	66.1 ± 13.2
VL MdPF (Hz)	71.4 ± 15.8	68.8 ± 16.6^a^	67.9 ± 13.6	68.1 ± 16.2	71.4 ± 12.1	69.2 ± 12.7	65.5 ± 11.0	65.9 ± 12.5
Average MdPF (Hz)	71.1 ± 16.0	69.3 ± 16.4	67.7 ± 13.6	68.4 ± 15.6	71.8 ± 11.8	69.8 ± 12.3	65.3 ± 10.8	66.0 ± 12.8
VM Burst (ms)	375.6 ± 127.2	363.0 ± 130.0	377.9 ± 196.2	317.6 ± 83.8	372.8 ± 102.7	322.0 ± 73.8^a^	367.5 ± 85.1	319.2 ± 72.1^a^^¶^
VL Burst (ms)	381.7 ± 120.6	321.3 ± 83.7^a^	343.6 ± 109.4	309.1 ± 73.1^T^	366.7 ± 107.3	315.0 ± 72.4^T^	371.4 ± 97.1	309.3 ± 60.0^a^^¶^
Average Burst (ms)	378.6 ± 113.0	342.2 ± 92.2^T^	360.8 ± 150.4	313.3 ± 77.6	369.8 ± 104.6	318.5 ± 58.9^a^	369.5 ± 88.6	314.2 ± 63.9^a^^¶^
VM Timing (%)	49.1 ± 3.4	46.3 ± 7.7	48.2 ± 3.5	48.1 ± 4.4	48.6 ± 3.0	47.8 ± 3.6	49.4 ± 3.4	47.4 ± 4.3^a^^¶^
VL Timing (%)	49.5 ± 3.6	50.2 ± 2.2	49.4 ± 2.5	49.7 ± 2.1	50.4 ± 1.6	49.4 ± 2.4	50.5 ± 2.3	49.4 ± 2.8
Average Timing (%)	49.3 ± 3.0	48.3 ± 4.2	48.8 ± 2.7	48.9 ± 3.0	49.5 ± 2.1	48.6 ± 2.8^T^	50.0 ± 2.8	48.4 ± 3.4^a^^¶^

*F_I_O_2_, inspiratory oxygen fraction; SpO_2_, hemoglobin saturation in capillary blood measured by pulse oximetry; Wmax, power output at exhaustion; VO_2_, oxygen consumption; V_E_, pulmonary ventilation; RR, respiratory rate; HR, heart rate; P_ET_O_2_, end-tidal O_2_ pressure; P_ET_CO_2_, end-tidal CO_2_ pressure; RER, respiratory exchange ratio; VCO_2_, CO_2_ production; RPM, revolutions per minute; VL, vastus lateralis; VM, vastus medialis; RMSraw, raw root mean square; RMSNz, normalized root mean square; TAINz: normalized total activation index (arbitrary units, A.U.); MPF, mean power frequency; MdPF, median power frequency; Burst: contraction burst duration; Timing, start of activation expressed as percentage of total revolution duration*.

a *P < 0.05*;

b *P < 0.01*;

c P < 0.001; and

T *P < 0.1 (F_I_O_2_ = 73 mmHg vs. new gas mixture)*.

¶ *P < 0.05 ANOVA breathing gas switch main effect*;

§ *P < 0.05 ANOVA oxygenation level main effect*;

‡ *P < 0.05 ANOVA breathing gas switch x oxygenation level interaction: n = 10*.

### Transition from severe hypoxia (P_I_O_2_ of 73 mmHg) to higher levels of P_I_O_2_

VL and VM RMSraw, RMSNz and TAINz were all enhanced by increasing the P_I_O_2_ at exhaustion (ANOVA main breathing gas effect *P* < 0.05) (Table 2). VM and VL RMSraw, as well as the VM-VL average RMSraw, were increased by 5–10% when the P_I_O_2_ was raised from 73 to 92, or 99 mmHg (Table 2). VL RMSraw and the VM-VL average RMSraw were also increased when the P_I_O_2_ was raised from 73 to 142 mmHg (Table 3). MPF and MdPF remained at the same level with the increase of P_I_O_2_.

### Transition to normoxia

As depicted in Table 3, increasing P_I_O_2_ from different hypoxia conditions to normoxia was also associated to increased VM and VL RMSraw and RMSNz, as well at VM TAINz and VM-VL Average TAINz (ANOVA breathing gas main effect *P* < 0.05) (Table 3). When the data from the two conditions with greater levels of hypoxia (P_I_O_2_ of 73 and 82 mmHg) were averaged, increasing P_I_O_2_ at exhaustion to normoxia significantly increased MA (RMSraw and RMSNz) and the normalized TAI (*P* < 0.05). However, this was not the case when the data from the less hypoxic conditions (P_I_O_2_ of 92 and 99 mmHg) were averaged, for which the transition to higher P_I_O_2_ did not result in significantly greater muscle activation. In general, MPF and MdPF remained at the same level or changed slightly with the transition to an increased P_I_O_2_.

We also analyzed the 10 s comprised between the 5th and the 15th second after the start of the transition and compared these 10 s with the last 10 s of the preceding exercise phase. The results of this analysis were essentially similar to those described above, i.e., increasing P_I_O_2_ at exhaustion resulted in increased MA (RMSraw and RMSNz), particularly when fatigue occurred at high levels of hypoxia (P_I_O_2_ of 73 and 82 mmHg).

In general, the pedaling rate was augmented with increased oxygenation at the transition from different levels of hypoxia to normoxia, and consequently, the duration of the contraction bursts was reduced (Table 3). At the same time, the start of the contraction bursts occurred slightly earlier with an increase in oxygenation from a P_I_O_2_ of 73 mmHg to normoxia.

### The first transition compared with the second transition

**Figure 4:**
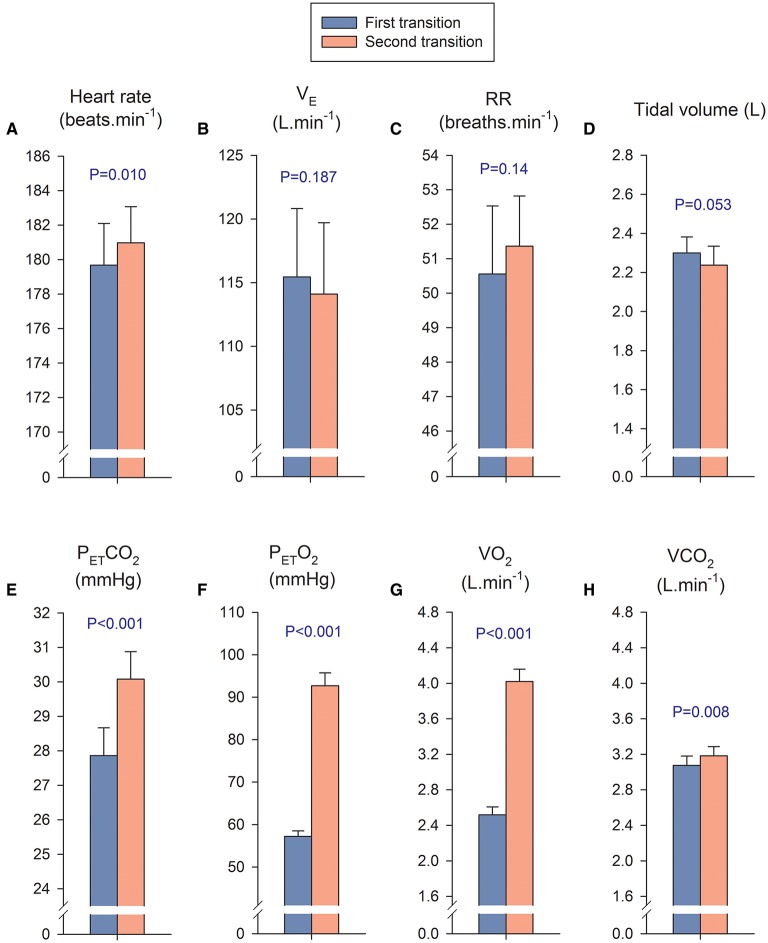
**Cardiorespiratory variables during the first and second transition**. In the first transition, the P_I_O_2_ was increased from severe hypoxia (P_I_O_2_ = 73 mmHg) to less hypoxic levels, while during the second transition the P_I_O_2_ was increased from different levels of hypoxia to normoxia. We calculated a mean value for the four P_I_O_2_ conditions of the first transition (blue bars) and compared it with the mean value of the four conditions of the second transition (orange bars), including in the analysis only the breath-by-breath data collected during the first 10 s of each transition. The mean response of the first transition was compared with the mean response of the second transition with a Student's paired *t*- test. It is important to remark that in both transitions the absolute exercise intensity was similar (170–173 W), however there is a remarkable difference in VO_2_ which is explained by the massive passage of O_2_ from the alveoli to the lung capillaries, driven by the much higher P_ET_O_2_ during the second transition. This massive diffusion of O_2_ is facilitated by the low SaO_2_ of the hemoglobin arriving to the lung capillaries at maximal exercise in hypoxia (Calbet et al., 2016). **(A)** Heart rate; **(B)** B pulmonary ventilation (V_*E*_); **(C)** respiratory rate (RR); **(D)** tidal volumen; **(E)** end-tidal CO_2_ pressure (P_ET_CO_2_); **(F)** end-tidal O_2_ pressure; (P_ET_O_2_), **(G)** Oxygen uptake (VO_2_); **(H)** CO_2_ production (VCO_2_). Each bar corresponds to the mean of 10 subjects; error bars represent the standard error of the mean.

In the first transition, the P_I_O_2_ was increased from severe hypoxia (P_*I*_O_2_ = 73 mmHg) to less hypoxic levels, while during the second transition the P_I_O_2_ was increased from different levels of hypoxia to normoxia. We calculated a mean value for the four P_I_O_2_ conditions of the first transition and compared it with the mean value calculated using the four conditions of the second transition, including in the analysis only the breath-by-breath data collected during the first 10 s of each transition. The mean P_I_O_2_ during the first and second transition was 84.3 ± 2.1, and 137.5 ± 3.0 mmHg, respectively, (*P* < 0.001); while SpO_2_ was 64.1 ± 4.8 and 72.0 ± 4.7%, respectively, (*P* < 0.001). The mean exercise intensity at which the first and second transitions occurred was 170.5 ± 17.9 and 173.5 ± 16.3 W (*P* = 0.08). The mean response of heart rate, pulmonary ventilation, respiratory rate and tidal volume were similar in both transitions (Figures 4A–D, respectively). In contrast, the P_ET_CO_2_, P_ET_O_2_, VO_2_, and VCO_2_ were higher during the second transition (Figures 4E–H, respectively).

**Figure 5:**
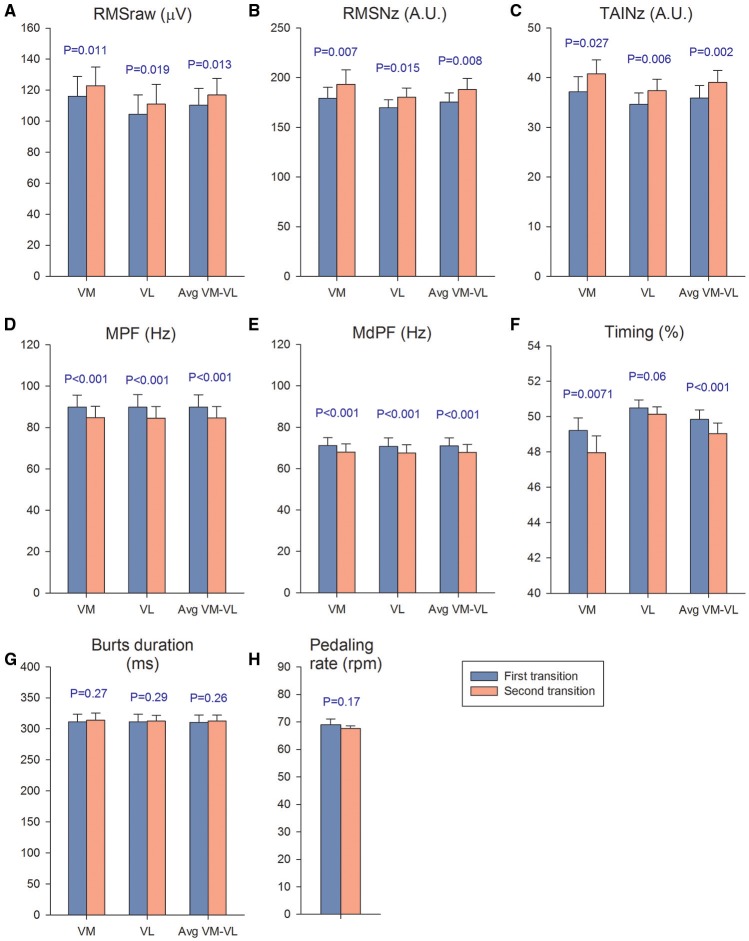
**Muscle activation and pedaling rate during the first and second transitions**. We calculated a mean value for the four P_I_O_2_ conditions of the first transition (blue bars) and compared it with the mean value calculated using the four conditions of the second transition (orange bars). The mean response of the first transition was compared with the mean response of the second transition with a Student's paired *t*-test. VM, *vastus medialis*; VL, *vastus lateralis*; **(A)** RMSraw, raw root mean square; **(B)** RMSNz, normalized root mean square; **(C)** TAINz, normalized total activation index; **(D)** MPF, mean power frequency; **(E)** MdPF, median power frequency; **(F)** Timing, start of activation expressed as percentage of total revolution duration; **(G)** burts duration; **(H)** pedaling rate. Each bar corresponds to the mean of 10 subjects; error bars represent the standard error of the mean.

Muscle activation was 6% higher during second compared to the first transition, as reflected by the VM, VL, and VM-VL average RMSraw values (*P* < 0.05) (Figure 5A). Similar results were obtained for the VM and VM-VL average RMSNz, which were 8 and 7% higher during the second compared to the first transition, respectively (*P* < 0.05) (Figure 5B). The VM, VL, and VM-VL average TAINz values were 8-10% higher during the second than the first transition (*P* < 0.05) (Figure 5C). VM, VL, and VM-VL average mean and median power frequencies were 4–6% lower during the second than the first transition (*P* < 0.001) (Figures 5D and E). The start of the burst occurred slightly earlier in the pedaling cycle during the second compared to the first transition for the VM and VM-VL average values, respectively, (*P* < 0.05) (Figure 5F). The duration of the burst and the mean pedaling rates were similar during both transitions (*P* > 0.56) (Figures 5G and H).

### Importance of the magnitude of the change in P_I_O_2_ and the pre-existing level of hypoxia on the response to an increase in P_I_O_2_

**Figure 6:**
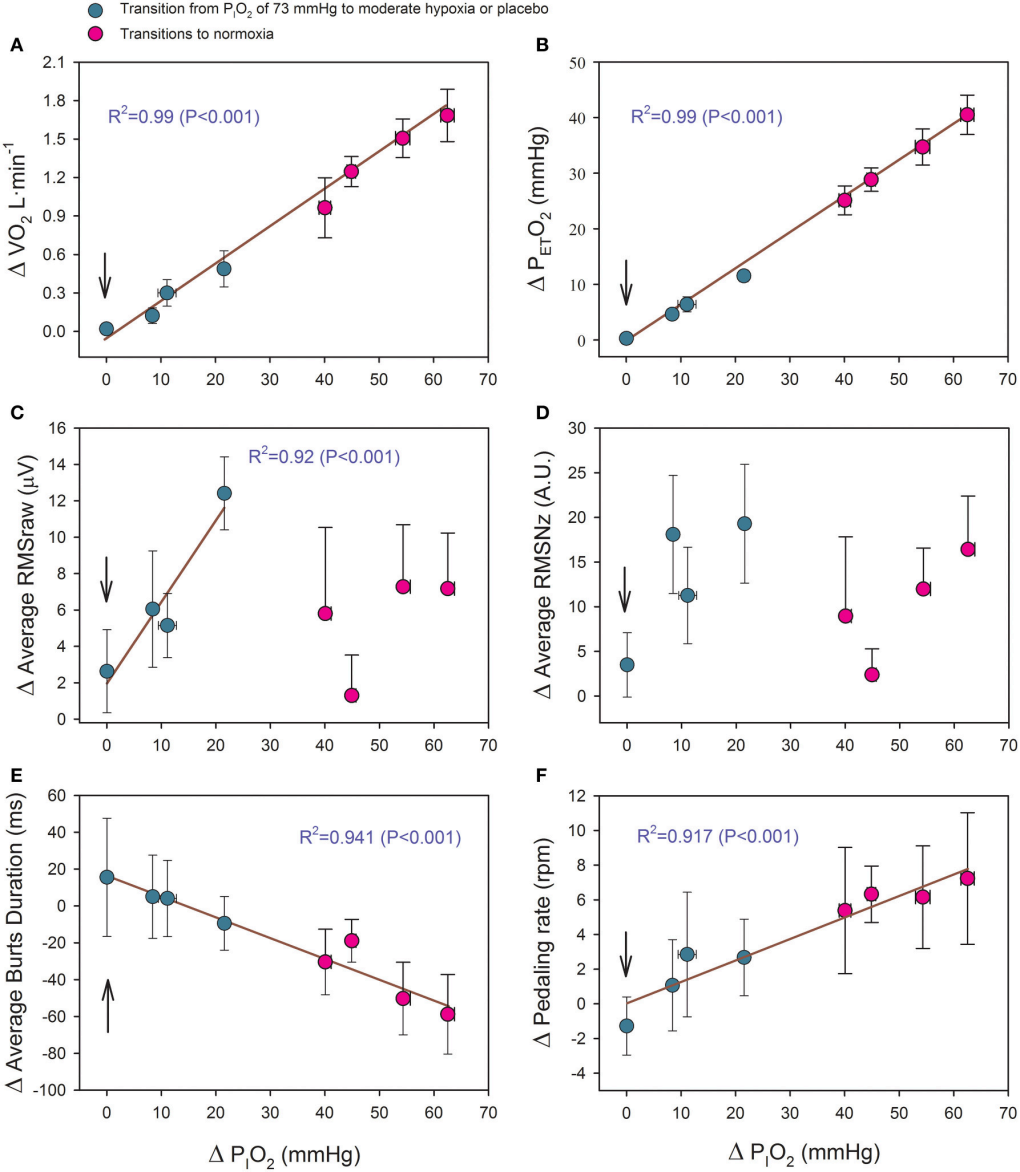
**Relationship between the magnitude of the P_**I**_O_**2**_ change (in mmHg) in transition from hypoxia to a higher P_**I**_O_**2**_ and the increases of: (A) oxygen uptake (VO_**2**_), (B) end-tidal O_**2**_ pressure (P_***ET***_O_**2**_), (C) average raw root mean square (RMSraw) of ***vastus medialis*** and ***vastus lateralis***, (D) average normalized root mean square (RMSNz) of ***vastus medialis*** and ***vastus lateralis***, (E) average burst duration of ***vastus medialis*** and ***vastus lateralis***, and (E) pedaling rate**. The vertical arrow indicates the placebo condition. Green circles: transitions from severe hypoxia (P_I_O_2_ = 73 mmHg) to placebo (vertical arrow) and moderate hypoxia; red circles: transitions from different levels of hypoxia (P_I_O_2_ of 73, 82, 92, and 99 mmHg) to normoxia. Each point corresponds to the mean of 10 subjects; error bars represent the standard error of the mean.

As reflected in Figure 6, the changes of P_ET_O_2_, VO_2_, the duration of the bursts and pedaling rate (PR) were linearly related to the increase in P_I_O_2_ as shown in the equations:

Equation 1 $$\begin{array}{l} \Delta {\text{VO}}_{2}=0.0277\;\cdot \;\Delta {\text{P}}_{\text{I}}O_{2}-0.0514({\text{R}}^{2}=0.990;\\ \text{ P}<0.001;\text{n}=8); \end{array}$$,

(Figure 6A)

Equation 2 $$\begin{array}{l} \Delta {\text{P}}_{\text{ET}}{\text{O}}_{2}=0.654\;\cdot \;\Delta {\text{P}}_{\text{I}}{\text{O}}_{2}-0.852({\text{R}}^{2}=0.997;\\ \text{ P}<0.001;\text{n}=8); \end{array}$$,

(Figure 6B)

Equation 3 $$\begin{array}{l} \Delta \text{BD}=16.33-1.127\;\cdot \;\Delta {\text{P}}_{\text{I}}O_{2}(R^{2}=0.941;\\ \text{ P}<0.001;\text{n}=8); \end{array}$$,

(Figure 6E)

Equation 4 $$\begin{array}{l} \Delta \text{PR}=0.082-1.122\;\cdot \;\Delta {\text{P}}_{\text{I}}O_{2}({\text{R}}^{2}=0.917;\\ \text{ P}<0.001;\text{n}=8); \end{array}$$,

(Figure 6F)

Where ΔVO_2_ is expressed in L·min^−1^; ΔP_ET_O_2_ and ΔP_I_O_2_ in mmHg; BD in ms, and PR in rpm.

The VM-VL average RMSraw was linearly related to the increase in P_I_O_2_, but only in the transitions from a P_I_O_2_ of 73 mmHg to a higher P_I_O_2_ [Δ RMSraw (μV) = 1.945 + 0.449 · ΔP_I_O_2_ (*R*^2^ = 0.915; *P* < 0.05, *n* = 4)] (Figure 6C). This relationship was lost after normalization of the RMS (Figure 6D).

### Placebo effects

In the placebo transition, subjects believed that they were receiving normoxia upon exhaustion in severe hypoxia; however, they were maintained at the same level of hypoxia. No significant changes were observed in MA (RMSNz and TAINz) as a consequence of this placebo treatment (Table 2).

## Discussion

This study shows that MA during the last 10–30 s of an IE to exhaustion is lower in SAH than in normoxia, while at exhaustion in moderate hypoxia MA was similar to that observed at exhaustion in normoxia. We have shown that during exercise at different levels of hypoxia, increasing P_I_O_2_ at exhaustion with normoxic or less hypoxic gas mixtures rapidly relieves fatigue and allows for the continuation of exercise. This effect is accompanied by increased MA only when the level of hypoxia during the exercise eliciting exhaustion was severe (P_I_O_2_ of 73 mmHg, equivalent to an altitude close to 5200 m) and the P_I_O_2_ was increased to 92 mmHg or higher and the estimated SaO_2_ to 70% or higher. Nevertheless, the close linear relationship between the increase in MA (average of VM and VL RMSraw) and the increase in P_I_O_2_ (Figure 6C) indicates that during exercise in SAH any small increase in P_I_O_2_ could have a positive effect on muscle activation. This is also supported by the fact that during the first 10 s of the transitions, MA was higher during the second than the first transition, despite the fact that both transitions occurred at comparable exercise intensities. Moreover, our investigation has also demonstrated that an increase in MA after the increase of P_I_O_2_ at fatigue in hypoxia is not indispensable for the ergogenic effects elicited by the increase of P_I_O_2_. Collectively, our results suggest that severe hypoxia depresses the capacity of the central nervous system to activate the musculature during whole-body exercise to exhaustion, by a mechanism that can be swiftly reversed by increasing the P_I_O_2_.

### Severe hypoxia reduces the level of muscle activation attainable during incremental exercise to exhaustion

In support of a central predominance of task failure mechanisms is the rapid relief of fatigue with the increase of P_I_O_2_, e.g., when subjects at exhaustion are asked to continue the exercise once the hypoxic gas mixture they are breathing is swiftly switched to normoxic room air (Calbet et al., 2003a) or hyperoxic gas (Amann et al., 2007). This concurs with the demonstration of a greater functional reserve at task failure in SAH than in normoxia (Amann et al., 2007; Calbet et al., 2015a; Morales-Alamo et al., 2015; Torres-Peralta et al., 2016). However, increased P_I_O_2_ does not relieve fatigue when administered at exhaustion during whole-body exercise in moderate hypoxia (F_I_O_2_ = 0.15, equivalent to 2700 m above sea level) (Amann et al., 2007) or during exercise recruiting a small muscle mass in severe hypoxia (Calbet and Lundby, 2009).

It has been reported that a greater level of supraspinal fatigue occurs at task failure during whole-body (Goodall et al., 2010) and knee-extension exercise (Goodall et al., 2010) in hypoxia than in normoxia. This effect is more accentuated with increased severity of hypoxia (Goodall et al., 2010). Nevertheless, in contrast to our observations, quadriceps MA (EMG_*RMS*_) declined during repeated isometric muscle contractions (60% of the maximal voluntary contraction, 5 s/5 s contraction/recovery) to similar levels in severe hypoxia (F_I_O_2_ = 0.10) and in normoxia (Goodall et al., 2010). A crucial difference between whole-body and small muscle mass (knee extension) exercise in hypoxia is that for a given P_I_O_2_, pulmonary gas exchange is more perturbed during whole-body than small muscle mass exercise, as reflected by the larger alveolo-arterial O_2_ difference (A-aDO_2_) observed during whole-body compared to small muscle mass exercise (Calbet et al., 2009b). The larger A-aDO_2_ combined with a greater right-shift of the ODC during whole-body exercise in severe hypoxia causes more desaturation for a given PaO_2_ during whole-body than small muscle exercise in severe hypoxia (Calbet et al., 2009b). Consequently, with an F_I_O_2_ close to 0.10, SpO_2_ at exhaustion was 78% during knee extension exercise in a study by Goodall et al. (2010) and 63% in the current investigation (Calbet et al., 2015a). In the present experiments, SpO_2_ was 79% at exhaustion when the P_I_O_2_ was 99 mmHg, a level of hypoxemia for which acute oxygenation at exhaustion did not enhance muscle activation. Although no definitive conclusion on which variable, PaO_2_ or SaO_2_, plays a more important role in the reduction of MA during exercise in severe hypoxia, our data combined with those of Goodall et al. (2010) indicate that MA is lower at exhaustion in hypoxia than in normoxia when the levels of SaO_2_ fall below ~70%, regardless of P_I_O_2_.

### Mechanisms by which hypoxia could reduce muscle activation close to exhaustion

Hypothetically, hypoxia could attenuate MA through two main mechanisms. Severe hypoxia could trigger inhibitory feedback at spinal and supraspinal levels reducing the discharge rate of spinal motoneurons compared to normoxia. Alternatively, severe hypoxia could limit or reduce the recruitment of high-threshold motor units. Regarding the first mechanism, animal studies have shown that levels of PaO_2_ similar to those observed in this investigation at exhaustion in SAH (Calbet et al., 2015a) increase the baseline discharge frequency of group III and especially of group IV muscle afferents in resting cats (Hill et al., 1992; Lagier-Tessonnier et al., 1993) and rabbits (Arbogast et al., 2000). Increased firing rates by group III/IV muscle afferents may cause reflex inhibition of the α-motoneuron pool (for review see Amann and Kayser, 2009) and, hence, reduced muscle activation. In the present investigation, MPF and MdPF were lower during the second that during the first transition (Figures 6D and F), coinciding with a greater attenuation of fatigue, likely due to the almost 5 times greater average ΔP_I_O_2_ during the second than the first transition. This finding does not necessarily indicates a change in motor activation, since MPF and MdPF are poor indices of motor unit recruitment patterns (Farina et al., 2014), and MPF and MdPF may be affected by peripheral factors, as recently demonstrated (Torres-Peralta et al., 2016). In agreement with our interpretation, increased metaboreflex activation during ischemic intermittent isometric muscle knee-extension contractions to exhaustion had no clear inhibitory effects on EMG_RMS_ values (Millet et al., 2009).

Regarding the second mechanism, hypoxia may reduce the oxygenation of the prefrontal, premotor, and motor cortex leading to a mismatch between energy demand and aerobic ATP re-synthesis, which could limit the corticospinal motor drive (Rasmussen et al., 2007; Verges et al., 2012). In agreement with this idea, our subjects developed a lower mean power output during the first 10 s of sprint exercise (30 s Wingate test) in severe hypoxia (P_I_O_2_ = 73 mmHg) than in normoxia, despite the fact that leg VO_2_ measured by the direct Fick method, was similar regardless of F_I_O_2_ (Calbet et al., 2015a). Interestingly, the duration of the contraction bursts was reduced and the start of the contraction bursts slightly advanced in the pedaling cycle with the transition from exhaustion in severe hypoxia to exercise in normoxia (Table 3). Likewise, subjects increased their pedaling rate in response to an increase of P_I_O_2_ at exhaustion. Interestingly, this effect was linearly dependent on the increase in P_I_O_2_ (Figure 6F). The latter implies that enhanced oxygenation (P_*a*_O_2_ and/or S_*a*_O_2_) at exhaustion swiftly alters the pattern of muscle activation/recruitment, likely allowing for a greater recruitment of faster motor units (Holt et al., 2014). This also points toward a central regulatory mechanism. In contrast, there was no improvement in any variable during the placebo transition, indicating that the level of oxygenation (P_a_O_2_ and/or S_a_O_2_) and the central command barely changed. Had the placebo transition reduced the perception of effort, a change would have been expected in the cardiorespiratory response to exercise (Robertson, 1982; Calbet et al., 2015b; Cochrane et al., 2015). Since all subjects believed that they were receiving oxygen-enriched gas at all transitions, included the placebo transition, and no subject was able to guess whether a gas mixture other than normoxia was administered, we can rule out psychological factors as being responsible for the changes in MA elicited by increased P_I_O_2_. In turn, psychological factors likely explain the ~42 additional seconds that the subjects were able to exercise during the placebo transition.

Although iEMG increases with increasing angular velocity during concentric contractions (Westing et al., 1991; Amiridis et al., 1996), this factor alone cannot explain the increased MA elicited by increased P_I_O_2_ in our experiment. In fact, pedaling rate did not increase significantly in the transition from severe hypoxia to moderate hypoxia (i.e., P_I_O_2_ of 92, and 99 mmHg), while MA was increased.

### Limitations

Although the amplitude of the surface EMG signal can provide a useful approximation of the amplitude component of the neural drive to muscle during some controlled conditions including dynamic exercise (Farina et al., 2014; Coelho et al., 2015), it has limitations. For example, the EMG signal is affected by the thickness of the subcutaneous adipose tissue, the spatial resolution is low overrepresenting superficial muscles fibers, may be altered by cross-talk from neighboring muscles, and is affected by the electrical properties of the sarcolemma, which may change during exercise (Farina et al., 2014). None of these factors is expected to change much within the first 10 s of increased P_I_O_2_ in our experimental conditions because exercise intensity was maintained at the same level during the first 2 min following the change in P_I_O_2_. The fact that the EMG amplitude of an interference signal is less than that obtained by summing the amplitudes of the individual motor unit action potentials, a phenomenon referred to as amplitude cancelation, also limits the interpretation of our results. Amplitude cancelation increases monotonically as the neural drive to muscle is elevated, affecting mostly the low-threshold motor units (Mottram et al., 2005; Farina et al., 2014). In our experimental conditions, reduced amplitude cancelation during the first 10 s of the transitions from task failure to increased P_I_O_2_, as a mechanism to explain the increase in EMG amplitude, is also unlikely since increasing P_I_O_2_ is expected to facilitate the neural drive to the muscles. To circumvent these limitations we have focused on assessing changes during the last 10 s of a given hypoxic condition and the first 10 s of the change to a higher P_I_O_2_. With such a short period, and given the stability of load at the start of the transition, the metabolic changes in the muscles should have been minuscule. This minimized the potential alteration of the EMG due to modification of the electrical properties of the muscle fibers and local metabolic factors during the first 10 s of the transition to a higher P_I_O_2_.

Another limitation of this study is due to the use of finger pulse oximetry rather than the direct assessment of SaO_2_, which is less accurate at SaO_2_ below 75–85% (Trivedi et al., 1997; Kolb et al., 2004). Another drawback of finger pulse oximetry is related to the slow response time of all pulse oximeters (Trivedi et al., 1997), with the delay being higher for finger than earlobe placements (Trivedi et al., 1997; Hamber et al., 1999). Consequently, the SpO_2_ values recorded during the first 10 s of the transitions from hypoxia to higher P_I_O_2_ underestimated the actual SaO_2_ values. Nevertheless, we should emphasize that the readings of our pulse oximeter were closely correlated to the SaO_2_ values, when measured simultaneously under steady conditions in the same subjects included in this study (SaO_2_ = 1.005 × SpO_2_ – 0.76, *n* = 74, SaO_2_ range: 53.9–96.5%, *R*^2^ = 0.99). Since during the transitions VO_2_ increased linearly with the increase of P_I_O_2_ (Figure 6A), oxygen transport from the lungs to the muscles and the central nervous system must have also been enhanced.

In summary, this investigation demonstrates that close to task failure, MA is lower during IE test to exhaustion in SAH than in normoxia. We have shown that increasing P_I_O_2_ at exhaustion reduces fatigue and allows for the continuation of exercise in moderate and severe acute hypoxia, regardless of the effects of oxygenation on muscle activation. In hypoxia, MA at task failure is increased within 10 s of oxygenation when task failure occurred at levels of hypoxia equivalent to an altitude close to 5200 m above sea level (P_I_O_2_ ~ 73 mmHg), and when the P_I_O_2_ is increased to levels ≥ 92 mmHg and SaO_2_ above 70%. Overall, these findings indicate that one of the central mechanisms by which severe hypoxia may cause central fatigue and task failure is by reducing the capacity for maximal muscle activation. The fact that exercise could be continued at exhaustion in severe hypoxia with the administration of a placebo-gas mixture demonstrates that this central mechanism has a cognitive component.

## Author contributions

Conception and design of the experiments: JC; pre-testing, experimental preparation, data collection, and analysis: RT, DM, JL, IP, JP, and JC; EMG analysis: RT, MG, and MI. The first version of the manuscript was written by RT and JC. All co-authors read and approved the final version of the manuscript.

### Conflict of interest statement

The authors declare that the research was conducted in the absence of any commercial or financial relationships that could be construed as a potential conflict of interest.
